# The value of modified Ross score in the evaluation of children with severe lower respiratory tract infection admitted to the pediatric intensive care unit

**DOI:** 10.1007/s00431-022-04737-9

**Published:** 2022-12-06

**Authors:** Enas Saad Hassan, Salah-Eldin Amry Ahmad, Ismail Lotfy Mohamad, Faisal-Alkhateeb Ahmad

**Affiliations:** Pediatric Department, Faculty of Medicine, Assiut University, Assiut, 71515 Egypt

**Keywords:** Heart failure, Lower respiratory tract infection, Modified Ross score, Pediatric

## Abstract

Heart failure (HF) represents an important cause of morbidity and mortality in children. It is mostly caused by congenital heart disease (CHD) and cardiomyopathy. The Ross HF classification was developed to assess severity in infants and has subsequently been modified to apply to all pediatric ages. The modified Ross classification for children provides a numeric score comparable with the New York Heart Association (NYHA) HF classification for adults. The aim of this work is to investigate the role of modified Ross score in the evaluation of children with severe lower respiratory tract infection admitted to the pediatric intensive care unit (PICU). One hundred and sixty-four children with severe LRTI admitted to the PICU were enrolled in this prospective cohort study, which was carried out at Assiut University Children Hospital, from the start of July 2021 up to the end of December 2021. Sixty patients (36.6%) of studied cases with severe LRTI admitted to PICU had HF. Out of these, 37 (61.7%) had mild HF; 17 (28.3%) had moderate HF, while six cases (10%) had severe HF according to the modified Ross score. The value of modified Ross score was significantly higher in children with heart failure with sensitivity and specificity 100% with cutoff value of 2. Admission to NICU, history of previous ventilation, and prematurity were higher in patients who developed HF. Patients with pulmonary hypertension (PH) and those with raised neutrophil lymphocyte ratio were significantly higher in the group of patients with moderate and severe degree of HF.

*Conclusion*: Modified Ross score is a simple clinical score which may help in assessing and predicting children with severe LRTI.

**Table:** 

**What is Known:** *• Hear failure is common complication to lower respiratory tract infection.* *• Modified Ross score was used to predict and classify heart failure in adult with lower respiratory infection.*	
**What is New:** *• Modified Ross score found to be of value in prediction of heart failure in children with lower respiratory tract infection.*

**What is Known:**

*• Hear failure is common complication to lower respiratory tract infection.*

*• Modified Ross score was used to predict and classify heart failure in adult with lower respiratory infection.*

**What is New:**

*• Modified Ross score found to be of value in prediction of heart failure in children with lower respiratory tract infection.*

## Introduction

Heart failure (HF) has been defined as an abnormality of cardiac structure or function leading to failure of the heart to deliver oxygen at a rate suitable for the requirements of the metabolizing tissues, despite normal filling pressures. HF in children is a progressive clinical and pathophysiological syndrome caused by cardiovascular and non-cardiovascular abnormalities that result in characteristic signs and symptoms including edema, respiratory distress, growth failure, and exercise intolerance [1]. Pediatric heart failure (PHF) is not common but devastating diagnosis affect in approximately 1 in 100,000 children worldwide [2].

The care of children suffering from HF presents unique challenge, regardless of the underlying etiology, PHF outcomes remain poor despite growing resource utilization. In addition, given the overlap in symptomatology between HF and more common childhood illnesses, the diagnosis of new onset PHF requires a heightened level of suspicion in combination with early pediatric cardiology consultation [3].

Until 1987, the only system available for grading HF in children was the New York Heart Association (NYHA) classification. However, this system was based on limitations to physical activity for adults, which did not translate well for use with children, particularly infants. It has been 25 years since the Ross classification was first used for this purpose. Since then, several modifications of the system have been used and others proposed [4].

The New York Heart Association (NYHA) HF classification is not readily applicable to younger children. Meanwhile, the modified Ross classification is used for HF severity classification in children [5].

Lower respiratory tract infections (LRTI), defined in the Global Burden of Diseases (GBD), Injuries, and Risk Factors Study as pneumonia or bronchiolitis, are a leading cause of mortality and morbidity worldwide. Nearly 2.38 million deaths resulted from lower respiratory infections in 2016, making lower respiratory infections the sixth leading cause of mortality for all ages and the leading cause of death among children younger than 5 years [6].

## Aim of the work

To assess the value of modified Ross score in detection of children at risk to develop heart failure associated with LRTI.

## Patients and methods

One hundred and sixty-four children with severe LRTI admitted to pediatric ICU were enrolled in this prospective cohort study, which was carried out at Assiut University Children Hospital, from the start of July 2021 up to the end of December 2021.

Age ranged from 1 month to 18 years old. Neonates; patients with heart, renal, liver, and/or hematological diseases; and those who refused to participate in this study were excluded.

LRTI was diagnosed according to revised WHO guidelines when a child had cough or difficulty breathing, with either lower chest wall indrawing (LCWI) or age-appropriate tachypnea (≥ 50 breaths/min if 2–12 months of age, ≥ 40 breaths/min if ≥ 12 months of age). Severe LRTI was diagnosed in children aged < 2 months with tachypnea > 60 breaths/min or LCWI, or in children of any age with danger signs (cyanosed, unable to drink, seizures, or decreased level of consciousness) [7].

All studied cases were subjected to full medical history, thorough examination, chest radiograph posterior-anterior view in erect position, full echocardiographic study, laboratory investigations including complete blood count, acute phase reactants, serum electrolytes or blood culture according to indications set by patient state, and evaluation of degree of HF was done using the modified Ross score for HF in children [4].

The total score of each child was calculated as follows: 0–2 (no hear failure), 3–6 (mild heart failure), 7–9 (moderate heart failure), 10–12 (severe heart failure). The main items evaluated were the history, physical examination, age, respiratory rate, heart rate, and hepatomegaly.

## Ethical issues

Confidentiality was maintained during all stages of the assessment, informed written consent was taken from parents of the children participating in the study, and approval of ethical committee of Assiut medical school was obtained (IRB no.: 17100686).

## Statistical analysis

Windows SPSS 22 was used in statistical analysis. Results were reported as mean ± standard deviation (SD) and number (percentage). Student’s *t*-test or Mann–Whitney *U* test was used for analyzing continuous variables. Chi-square test and Fisher exact test were used for categorical variables. *p* value < 0.05 was considered statistically significant. The area under curve (AUC), sensitivity, specificity, positive and negative likelihood ratios, and 95% confidence intervals were calculated for the cutoff values.

## Results

The median age of the studied participants was 5 months ranged from 2 months up to 17 years old with 66.5% of the cases below 1 year of age. Out of 164 patients, 88 (53.7%) were male with a male:female ratio of 1.2:1. Pediatric patients with previous history of NICU admission, those who received mechanical ventilation (MV), and preterm infants were more liable to develop HF than their counterpart. Patients with HF suffered from lower weight and height (< 5th centile) than those who did not develop HF (Table 1).

**Table 1:** Demographic data of studied group of patients according to presence of heart failure

Baseline data	Total cases (*n* = 164)		HF group (*n* = 60)		No HF group (*n* = 104)		*p* value
	No.	%	No.	%	No.	%	
Age (months)							
≤ 12	109	66.5%	32	53.3	77	74.0	0.007*
> 12	55	33.5%	28	46.7	27	26.0	
Sex							
Male	88	53.7%	28	46.7	60	57.7	0.173
Female	76	46.3%	32	53.3	44	42.3	
Previous NICU admission							
Yes	24	14.6%	24	40.0	0	0.0	< 0.001*
No	140	85.4%	36	60.0	104	100.0	
Previous ventilation							
Yes	24	14.6%	24	40.0	0	0.0	< 0.001*
No	140	85.4%	36	60.0	104	100.0	
Maturity							
Full-term	130	79.3%	36	60.0	94	90.4	< 0.001*
Pre-term	34	20.7%	24	40.0	10	9.6	
Weight grade							
Above 5th	134	81.7%	30	50.0	104	100.0	< 0.001*
Below 5th	30	18.3%	30	50.0	0	0.0	
Height grade							
Above 5th	158	96.3%	54	90.0	104	100.0	0.002*
Below 5th	6	3.7%	6	10.0	0	0.0	

Qualitative data are presented as *n* (%). Significance defined by *p* < 0.05

*HF* heart failure, *NICU* neonatal intensive care unit

Clinical and laboratory findings in studied cases according to presence of HF and modified Ross score were summarized in Table 2 which revealed that children with higher degree of respiratory distress, cyanosis, edema, hepatomegaly, tachycardia, diminished perfusion, and diaphoresis were significantly common in those with heart failure compared to children without HF. As regards the laboratory data, it was found that patients who developed HF suffered from higher leukocytic count and raised neutrophil/lymphocyte ratio (NLR) as compared to patients who did not developed HF. The same clinical and laboratory findings were found when we compared the cases according to the modified Ross score into mild versus moderate to severe.

**Table 2:** Clinical and laboratory findings in studied cases according to presence of HF and modified Ross score

Clinical data	Total cases (*n* = 164)		HF group (*n* = 60)		No HF group (*n* = 104)		*p* value	Degree of HF				*p* value
								Mild cases (*n* = 37)		Moderate/severe (*n* = 23)		
	No.	%	No.	%	No.	%		No.	%	No.	%	
RD grade							< 0.001*					
Grade I	74	45.1%	25	41.7	49	47.1		22	59.5	3	13.0	
Grade II	67	40.9%	18	30.0	49	47.1		12	32.4	6	26.1	< 0.001*
Grade III	18	10.9%	12	20.0	6	5.8		3	8.1	9	39.1	
Grade IV	5	3.0%	5	8.3	0	0.0		0	0.0	5	21.7	
Cyanosis	5	3.0%	5	8.3	0	0	0.006*	0	0.0	5	21.7	0.006*
Edema	6	3.7%	6	10	0	0	0.002*	1	2.7	5	21.7	0.027*
Hepatomegaly	24	14.6%	24	40	0	0	< 0.001*	9	24.3	15	65.2	0.002*
Tachycardia for age	51	31.1%	51	85.0	0	0.0	< 0.001*	29	78.4	22	95.7	0.134
Diminished perfusion	7	4.3%	7	11.7	0	0.0	0.001*	0	0.0	7	30.4	0.001*
Diaphoresis	30	18.3%	30	50.0	0	0.0	< 0.001*	11	29.7	19	82.6	< 0.001*
Prognosis												
Good (discharge)	154	93.9%	50	83.3	104	100.0	< 0.001*	37	100.0	13	56.5	< 0.001*
Poor (death)	10	6.1%	10	16.7	0	0.0		0	0.0	10	43.5	
Laboratory findings												
NLR	23	14.0%	23	38.3	0	0.0	< 0.001*	1	2.7	22	95.7	< 0.001*
Leukocytosis	148	90.2%	60	100.0	88	84.6	0.001*	37	100.0	23	100.0	–
CRP	123	75.0%	49	81.7	74	71.2	0.134	30	81.1	19	82.6	1.000

Qualitative data are presented as *n* (%). Significance defined by *p* < 0.05

*HF* heart failure, *RD* respiratory distress, *CRP* C-reactive protein

Also, Table 2 shows that children with LRTI who developed HF had poorer prognosis (16.7% died) as compared to patients who did not developed HF (*p* < 0.001); the same findings were observed when we compared cases according to the modified Ross score (mild versus moderate to severe).

Patients with poor prognosis (died) had higher leukocytic count and raised NLR compared to patients with good prognosis (improvement and discharge from the pediatric ICU) (*p* < 0.001) as shown in Table 3.

**Table 3:** White blood cells and neutrophil lymphocytic ratio according to prognosis

	Good prognosis	Poor prognosis	*p* value
WBCs 10^9^ × /L			
Mean ± SD	10.49 ± 4.58	37.80 ± 6.36	< 0.001*
Median (IQR)	10.0 (7.0–13.0)	37.5 (33.0–42.0)	
NLR			
Mean ± SD	0.83 ± 0.69	3.05 ± 0.99	< 0.001*
Median (IQR)	0.5 (0.4–1.0)	2.9 (2.6–3.5)	

Quantitative data are presented as mean ± SD and median (range). Significance defined by *p* < 0.05

*WBCs* white blood cells, *NLR* neutrophil lymphocytic ratio

The value of modified Ross score in children with heart failure (6.15 ± 2.38), compared to children without heart failure (1.96 ± 0.80) with significant higher value in heart failure cases (*p* < 0.001) (Table 4).

**Table 4:** The value of modified Ross score in children with heart failure compared to those without heart failure

**MRS**	**Heart failure**	**No heart failure**	***p*** **value**
**Mean ± SD**	6.15 ± 2.38	0.96 ± 0.80	< 0.001*
**Median (IQR)**	6.0 (4.0–8.0)	1.0 (0.0–2.0)	

**Fig. 1:**
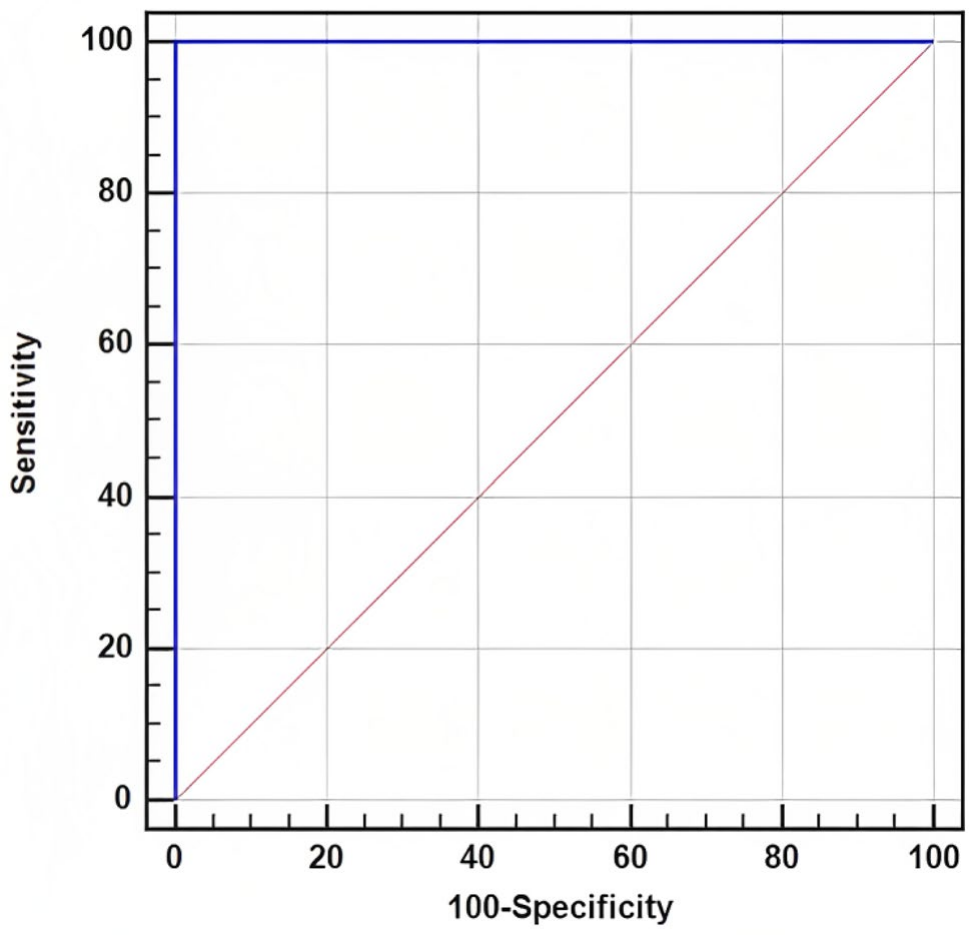
Modified Ross score sensitivity, specificity, and area under the curve

Figure 1 shows the sensitivity, specificity, and area under the curve of the value of modified Ross score in predicting heart failure in children with LRTI; it was 100% for both sensitivity and specificity with UAC equal to 1 when the cutoff value equal to 2.

## Discussion

The cardiovascular and respiratory systems function as a single unit and alteration in cardiorespiratory interactions can cause significant changes in cardiac function [8]. Studies in adult patients revealed that the presence of a cardiac event, such as congestive heart failure (CHF), is among the leading complications that increase the chances of morbidity and mortality among patients with LRTI [9, 10]. However, the association in pediatric patients is not well established especially in those with no underlying cardiac diseases. Present study also tried to explore the characteristics and factors associated with the development of HF in children reporting with LRTI and found the prevalence of heart failure (CCF) was 36.6%.

Few studies that have been conducted on the present matter have reported different prevalence of the disease; one study conducted in the pediatric wards of a tertiary hospital in Nigeria by Sadoh and Osarogiagbon reported that 64% of patients with congestive cardiac failure had pneumonia with underlying congenital heart disease (CHD) and 37% of patients with pneumonia without any underlying illness [11]. Also Ýlten and his colleagues found an incidence of congestive heart failure in 14% among 50 children with pneumonia [12], Navarro et al. reported HF in 8.5% of the studied cases [13], and Kabir et al. reported that 9 (25%) children developed heart failure [8]. And recently Jat et al. reported lower rate as the authors reported that out of 140 cases of pneumonia without CHD 28 (20.0%) had CCF [14].

Our reported rate of HF development among patients with LRTI was much higher than the rate reported by most of above mentioned studies [12–14]; this may be attributed to that we included all pediatric patients with LRTI, also we excluded patients with past history of CCH which is a known risk factor for developing LRTI [15].

Regarding demographic characteristics of the studied cases with severe LRTI with and without HF according to Ross classification, it was observed that the percentage frequency of cases under 12 months old was significantly higher than those above 12 months old. This could be due to the difference in etiology of respiratory disease between the two groups. Cases with severe lower respiratory infection due to bronchopulmonary dysplasia, interstitial pulmonary fibrosis, and bronchiectasis were only found in the group of patients who developed heart failure. These diseases are known to cause PH and secondary right heart affection [16].

It was observed that pediatric patients with previous history of NICU admission, those who received MV were more liable to develop HF than their counterpart. This could be explained by the etiology of the disease. All cases with bronchopulmonary dysplasia developed HF secondary to severe LRTI, and most of these cases require NICU admission and MV [17]. Also, patients with HF suffered from lower weight (< 5th centile) than those who did not develop HF, as growth failure may be the only manifestation of HF in young children [4]. In line with our study, Jayaprasad [1] stated that established HF presents with poor weight gain and in the longer term, failure in linear growth can also result.

Prematurity predicts severe viral LRTI and mortality in both low-income and high-income countries (HICs) according to Le Roux et al. [18]. This supports our finding as we observed that premature infants were more liable to develop severe acute LRTI and HF (according to modified Ross score) compared to full-term babies.

In the current study, we observed that pediatric patients who developed HF suffered from higher degree of respiratory distress, cyanosis, edema, hepatomegaly, tachycardia, diminished perfusion, and diaphoresis as compared to patients who did not developed HF. The same finding was found when we divide studied cases according to the modified Ross score into mild versus moderate to severe. This finding is also similar to those found in other studies [19, 20].

Jayaprasad [1] reported similar finding. He reported that tachycardia > 150/min, respiratory rate > 50/min, gallop rhythm, and hepatomegaly are features of HF in infants.

Similar result was obtained by Jat et al. [14] who stated that the most common symptoms for patients with congestive cardiac failure (CCF) were respiratory distress (84%), hepatomegaly (81%), feeding difficulty (79.4%), diaphoresis (50%), and poor weight gain (3.2%).

It was observed that cases with PH as detected by echocardiography were detected only among the group of patients with severe LRTI who developed HF according to Ross score (*p* < 0.001).

Bronchopulmonary dysplasia (BPD) disease which following preterm birth is a developmental lung disease that may result in persistent airway and pulmonary vascular morbidity in childhood. Recurrent hospitalization for respiratory decompensation, often in the setting of intercurrent infection, may be accompanied by evidence of elevated right heart pressures and pulmonary vascular resistance (PVR) [16]. Evidence shows that 18% of very low birth weight (VLBW) infants have some degree of PHT during hospitalization and this incidence increases to 25–40% in those with established BPD [21]. This comes in agreement with the present study as we found that 25.0% of studied cases with positive BPD versus 12.1% of studied cases with negative BPD developed PH. Also in accordance with our study, a recent meta-analysis of over 1400 patients from 25 publications confirmed the association of PH with severity of pulmonary disease with cumulative prevalence of PH in 6%, 12%, and 39% of infants with mild, moderate, and severe BPD, respectively [22].

In 2010 Bardi-Peti and Ciofu study which aimed to assess whether previously healthy infants with acute respiratory diseases develop elevated pulmonary artery pressures and to identify which type of disease is associated with PH, the authors observed an increased in the mean pulmonary pressures (> 25 mmHg) in subjects with clinically broncho obstructive disease (bronchiolitis, episodic wheezing, bronchopneumonia) vs. control (*p* < 0.05). Also the same authors reported that hospitalization was significantly longer in patients with pulmonary hypertension vs. normal PAP (*p* < 0.05) [23].

Hypoxemia is the most significant risk factor for all LRTI-related deaths. In pneumonia, the inflammation of the alveoli and interalveolar septum causes exudation of fluid into the alveoli and edema of the inter-alveolar septum. The resulting ventilation perfusion mismatch leads to hypoxia. Hypoxia causes pulmonary vascular vasoconstriction which raises the pulmonary arterial vascular pressure. The right side of the heart eventually fails when it cannot adequately pump against the pulmonary pressure [24]. Furthermore, heart failure may develop due to direct damage to the myocardium by circulating toxins and microorganisms, disruption of the balance between right ventricular afterload and preload due to hypoxemia, and increased pulmonary artery pressure (PAP) due to pulmonary vasoconstriction (PH) [25].

In the current study, we observed that pediatric patients with LRTI who developed HF have poorer prognosis (16.7% were died) as compared to patients who did not developed HF (no case was died); the same finding was observed when we divide studied cases according to the modified Ross score, as we found that patients with moderate to severe HF have poorer prognosis as compared to patients with mild HF. Our reported mortality rate was much higher than what was reported by Jat et al. [14] who reported that the mortality rate in pneumonia without CHD was 3.57%. This variation in results can be attributed to difference in the etiology of LRTI as the authors only include pneumonia cases; meanwhile, in our study the main cause of LRTI among our studied cases who developed HF and admitted to PICU was bronchopulmonary dysplasia (BPD), also in addition to difference in ICU capabilities and skills.

Regarding the laboratory finding among our studied cases, we found that 23 (14%) had raised NLR, 123 (75%) cases had positive CRP, and 148 (90.2%) cases had leukocytosis. By comparing these laboratory data between patients who developed HF or not, we observed that patients with LRTI who developed HF have significantly higher NLR and higher leukocytic count. Those patients also have poorer prognosis (died) as compared to patient with good prognosis (improvement and discharge from the pediatric ICU) (*p* < 0.001). Interestingly, by using the modified Ross classification we found that cases with moderate to severe degree of HF have significantly higher NLR. This finding highlights the need to use both NLR and modified Ross classification as prognostic markers for poorer prognosis among pediatric patients with LRTI as this scale also was found to be capable to predict patients with poorer prognosis who need urgent treatment to minimize mortality among those patients.

In concordance to our finding, the recent study of Şık et al. in 2020 reported that at admission, the total leukocyte count in died patients was higher compared to survived patients (*p* = 0.001). When the highest values obtained during hospitalization were evaluated, the total leukocyte count in died patients was again higher compared to survived patients (*p* = 0.001) [26].

Murray et al. [27] and Cherry [28] also found that a high leukocyte count was associated with an increased need for MV, severe PH, and mortality in patients hospitalized with a diagnosis of pertussis.

Nevertheless, the calculated NLR is a sensitive biomarker to reflect the balance between inflammatory response and immune status in patients [29]. Existing data suggested that NLR could be used as a marker for risk assessment in patients with acute [30] and chronic HF [31], and closely associated with increased mortality [30].

It has also been reported that, compared with healthy people, NLR was significantly increased in pneumonia, indicating that it can be used as predictors for the presence of pneumonia [32, 33]. Another study has shown that NLR is a good indicator in evaluating prognosis of various diseases, such as malignant tumors [34].

On the other hand, Wu et al. observed lower level of NLR in pneumonia children. However, the underlying cause of lower NLR level in pneumonia group than that of URTI has not been yet clear [35].

## Conclusion

Modified Ross score is a simple clinical score which may help in assessing and predicting children with severe LRTI.

## Data Availability

Available on reasonable request.

## Abbreviations

GBD: Global Burden of Diseases
HF: Heart failure
LRTI: Lower respiratory tract infection
NYHA: The New York Heart Association
PICU: Pediatric intensive care unit
